# Analysis of the *In Vivo* Transcriptome of Bordetella pertussis during Infection of Mice

**DOI:** 10.1128/mSphereDirect.00154-19

**Published:** 2019-04-17

**Authors:** Ting Y. Wong, Jesse M. Hall, Evan S. Nowak, Dylan T. Boehm, Laura A. Gonyar, Erik L. Hewlett, Joshua C. Eby, Mariette Barbier, F. Heath Damron

**Affiliations:** a Department of Microbiology, Immunology, and Cell Biology, West Virginia University, Morgantown, West Virginia, USA; b Vaccine Development Center, West Virginia University Health Sciences Center, Morgantown, West Virginia, USA; c Division of Infectious Diseases and International Health, School of Medicine, University of Virginia, Charlottesville, Virginia, USA; University of Michigan-Ann Arbor; University of Maryland School of Medicine; University of Georgia; National Animal Disease Center, ARS, USDA

**Keywords:** Bordetella pertussis, *in vivo* RNA sequencing, infection transcriptome, pertussis, transcriptomics, whooping cough

## Abstract

Bordetella pertussis causes the disease whooping cough through coordinated control of virulence factors by the *Bordetella* virulence gene system. Microarrays and, more recently, RNA sequencing (RNA-seq) have been used to describe *in vitro* gene expression profiles of B. pertussis and other pathogens. In previous studies, we have analyzed the *in vitro* gene expression profiles of B. pertussis, and we hypothesize that the infection transcriptome profile *in vivo* is significantly different from that under laboratory growth conditions. To study the infection transcriptome of B. pertussis, we developed a simple filtration technique for isolation of bacteria from infected lungs. The work flow involves filtering the bacteria out of the lung homogenate using a 5-μm-pore-size syringe filter. The captured bacteria are then lysed to isolate RNA for Illumina library preparation and RNA-seq analysis. Upon comparing the *in vitro* and *in vivo* gene expression profiles, we identified 351 and 255 genes as activated and repressed, respectively, during murine lung infection. As expected, numerous genes associated with virulent-phase growth were activated in the murine host, including pertussis toxin (PT), the PT secretion apparatus, and the type III secretion system. A significant number of genes encoding iron acquisition and heme uptake proteins were highly expressed during infection, supporting iron acquisition as critical for B. pertussis survival *in vivo*. Numerous metabolic genes were repressed during infection. Overall, these data shed light on the gene expression profile of B. pertussis during infection, and this method will facilitate efforts to understand how this pathogen causes infection.

**IMPORTANCE** *In vitro* growth conditions for bacteria do not fully recapitulate the host environment. RNA sequencing transcriptome analysis allows for the characterization of the infection gene expression profiles of pathogens in complex environments. Isolation of the pathogen from infected tissues is critical because of the large amounts of host RNA present in crude lysates of infected organs. A filtration method was developed that enabled enrichment of the pathogen RNA for RNA-seq analysis. The resulting data describe the “infection transcriptome” of B. pertussis in the murine lung. This strategy can be utilized for pathogens in other hosts and, thus, expand our knowledge of what bacteria express during infection.

## Abstract (precis)

*In vitro* growth conditions for bacteria do not fully recapitulate the host environment. RNA sequencing transcriptome analysis allows for the characterization of the infection gene expression profiles of pathogens in complex environments. Isolation of the pathogen from infected tissues is critical because of the large amounts of host RNA present in crude lysates of infected organs. A filtration method was developed that enabled enrichment of the pathogen RNA for RNA-seq analysis. The resulting data describe the “infection transcriptome” of B. pertussis in the murine lung. This strategy can be utilized for pathogens in other hosts and, thus, expand our knowledge of what bacteria express during infection.

## INTRODUCTION

Bordetella pertussis is a bacterial respiratory pathogen and the causative agent of whooping cough, otherwise known as pertussis. While whooping cough is a vaccine-preventable disease, the number of cases has risen during the past decade (1). Reemergence of pertussis is a multifactorial problem that highlights the need to redesign and improve current vaccination strategies. Whole-cell vaccines (WCV), made of formalin-inactivated bacteria, were first introduced in the 1940s in the United States. Following implementation of the WCV, efforts by numerous research teams led to the identification of B. pertussis major virulence factors, such as the adenylate cyclase toxin (ACT), pertussis toxin (PT) (2), and others. The identification of B. pertussis major virulence factors also included the discovery of the global regulatory system of virulence genes (*Bordetella* virulence gene [Bvg] system) (3) and of important surface adhesins such as filamentous hemagglutinin (FHA) (4), fimbriae (FIM) (5), and pertactin (PRN) (6). The DTaP acellular vaccine is composed of PT, FHA, and PRN antigens (7), whereas the Tdap formulation includes the FIM antigen. Since the implementation of the acellular vaccination schedule, an alarming rise in pertussis cases have been observed. Due to the reemergence of pertussis (8, 9), there is a need to develop a third-generation pertussis vaccine. We hypothesize that achieving a deeper understanding of B. pertussis during infection of model organisms will aid in the identification of new protective antigens.

Microarrays have been utilized to study B. pertussis gene expression in regard to the Bvg system (10, 11), epidemic strains (12), *ptxP3* linage strains (13), the iron starvation response (14), the RisA regulon (15), Hfq-directed virulence genes (16), and others. Many studies performed in Bordetella bronchiseptica have detailed the gene expression of clinical and lab-adapted strains in response to different growth conditions (17–22). All of the aforementioned studies have significantly enhanced our knowledge about genetic regulation in *Bordetella*. For example, microarray analysis defined the list of virulence-activated genes (VAGs) and virulence-repressed genes (VRGs), which are controlled by BvgAS (11). The BvgAS master regulatory system controls three phases of B. pertussis: the Bvg^−^, Bvg^i^, and Bvg^+^ phases. Virulence-repressed genes are expressed during the Bvg^−^ phase. The Bvg^i^ phase occurs when the BvgAS system is partially active, and it controls the expression of genes such as *fhaB*, *fim*, and *bvgAS*. In the Bvg^+^ phase, BvgAS is fully active and controls expression of virulence-activated genes such as PT and ACT and genes involved in the type III secretion system (23).

As RNA sequencing (RNA-seq) developed and became more readily available to the research community, it was used to further study the importance of gene expression and regulation in *Bordetella.* For example, Ahuja et al. used RNA-seq transcriptomic analysis to characterize the genetic regulation of the type III secretion system (24). We followed the work of Ahuja et al. and characterized the role of the RpoE sigma factor in the global gene expression profile of B. pertussis and observed inverse regulation of pertussis and adenylate cyclase toxins (25), suggesting the presence of non-BvgAS regulatory mechanisms. Recently, Moon et al. also employed RNA-seq analysis to more precisely define the BvgAS regulon (26). Furthermore, Lesne et al. teased out the distinct virulence ranges of B. pertussis governed by this system during murine respiratory tract infection using *bvgS* mutants (27). After identifying the unique infection profiles of each strain, they performed *in vitro* RNA-seq to characterize the effects of the mutations in *bvgS* and how they affect downstream gene expression (27). While *in vitro* RNA-seq analysis provides important insights about regulation of virulence, only one study has been performed to determine what B. pertussis expresses in the host (28). van Beek et al. used microarrays to study the *in vivo* gene expression of B. pertussis in the murine airway (28). Interestingly, van Beek et al. identified several Bvg repressed genes that were highly expressed in the murine host. The study also detailed that 30% of the genome was differentially expressed in a comparison between *in vitro* and *in vivo* conditions (28).

We aimed to develop a robust RNA-seq methodology for gene expression analysis of B. pertussis in the murine host in order to describe the infection transcriptome of this pathogen. We have previously performed dual RNA-seq of Pseudomonas aeruginosa in the murine lung (29). Dual RNA-seq characterizes both the host and pathogen transcriptomes simultaneously. When we attempted to perform dual RNA-seq with B. pertussis, we identified an insufficient number of reads, and it was not possible to precisely characterize the bacterial pathogen’s *in vivo* transcriptome (30). In this study, we describe a simple filtration method that allowed us to isolate bacteria to facilitate characterization of the *in vivo* transcriptome of B. pertussis by RNA-seq. The *in vivo* gene expression profile of B. pertussis did not mimic a known virulence gene expression profile as precisely as expected. Genes encoding proteins involved in multiple pathways of iron acquisition were highly expressed *in vivo*, highlighting the need of the pathogen to obtain iron during infection. Overall results from this study suggest that additional factors, independent from the *Bordetella* virulence gene system, are involved in the pathogenesis and survival of B. pertussis during infection. In addition, the technical advances described here for bacterial isolation from complex tissues will facilitate the design of future strategies to characterize gene expression of B. pertussis and other pathogens during infection.

## RESULTS

### Developing a protocol for *in vivo* transcriptomic analysis of B. pertussis with RNA-seq.

**FIG 1:**
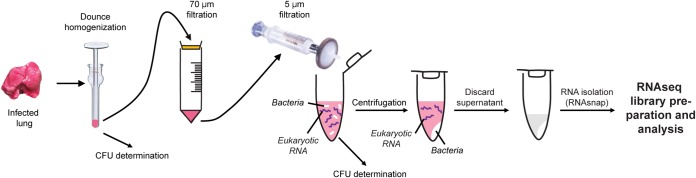
Experimental procedure for the isolation of bacterial RNA from infected tissue. Infected lungs were homogenized with a Dounce glass homogenizer, and the lung homogenate was filtered through a 70-μm-pore-size cell strainer to remove tissue debris. The strained homogenate was pushed through a 5-μm-pore-size syringe filter to exclude eukaryotic cells and capture the bacteria in the filtrate. The captured bacteria were pelleted by centrifugation, and the contaminating murine RNA (due to cell lysis) was removed. Once supernatant containing contaminating RNA was removed, the bacterial pellet was resuspended in RNAprotect to stabilize the bacterial cells until RNAsnap isolation.

**FIG 2:**
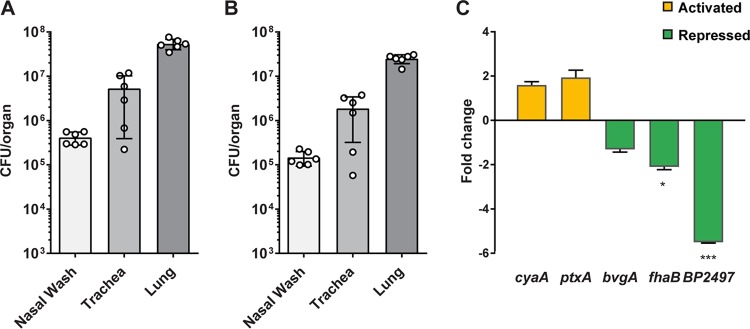
Isolation of B. pertussis and qRT-PCR analysis from infected NSG mouse lungs. NSG mice were infected by intranasal administration of B. pertussis strain UT25. At 3 days postinfection, the nasal wash, trachea, and lung were collected, and the numbers of input and output bacteria (postfiltration) were quantified. (A) Bacterial load in each tissue after homogenization. (B) Number of bacteria recovered from each tissue after implementing the filtration strategy. (C) qRT-PCR analysis was performed on B. pertussis strain UT25 grown in SSM broth and compared to RNA from the B. pertussis isolated from NSG lungs by the filtration protocol. Fold change in gene expression levels between *in vivo* and *in vitro* growth conditions is shown. Data are from three biological replicates with three technical replicates each. Standard deviations were calculated from variations between biological replicates and compared using a *t* test (*, *P < *0.05; ***, *P < *0.001).

Our previous studies with the respiratory pathogen P. aeruginosa demonstrated the feasibility of sequencing both bacterial and pathogen RNAs during infection (29). However, that study also highlighted the need to have high bacterial loads (10^8^ to 10^9^ CFU/organ) to obtain sufficient RNA for purification and analysis. We initially attempted to perform RNA-seq of B. pertussis infecting the trachea of outbred CD1 mice; however, those attempts resulted in insufficient bacterial reads for transcriptomic analysis (data not shown). We then identified the NSG (NOD.Cg-*Prkdc^scid^ Il2rg^tm1Wjl^*/SzJ) mouse as a highly susceptible strain due to a lack of mature lymphocytes caused by a *scid* mutation in the DNA repair complex protein *Prkdc* and a mutation in the interleukin-2 (IL-2) receptor common gamma chain, causing a deficiency in functional NK cells. Consequently, the NSG mouse also harbors defective macrophages and dendritic cells, with only neutrophils and monocytes unaffected by the mutations. With this in mind, we aimed to use the high bacterial burden in NSG mice to facilitate RNA-seq of infected murine lung (30). However, we were able to map only approximately 15,000 reads per 100 million to the B. pertussis genome (30). Despite the limitations of that data set, we observed that the alcaligin siderophore biosynthesis genes were highly expressed by B. pertussis infecting the NSG mouse lung (30). Furthermore, we saw that most reads mapped to *fhaB*, which encodes FHA, suggesting that this gene could be highly expressed during infection. In our previous study of the RpoE transcriptome *in vitro,* we observed that *fhaB* is one of the most abundant transcripts in B. pertussis (25). These data encouraged us to continue to refine and develop the *in vivo* RNA-seq work flow and methods. In order to maximize removal of host RNA, we used the small size of the bacterium as a means to separate it from the host cells. B. pertussis is approximately ∼1 µm in length by 0.5 µm in width (Fig. 1). Using *in vitro*-grown cultures, we determined that 5-µm-pore-size syringe filters (Minisart; Sartorius) allowed us to collect ∼50% of the viable bacteria in one step (Fig. 2A and B).

### Isolation and qRT-PCR analysis of B. pertussis from infected murine respiratory tissues.

We next aimed to harvest B. pertussis from infected NSG lungs at 3 days postinfection using the 5-µm-pore-size syringe filtration. The lungs were homogenized and plated on Bordet-Gengou (BG) agar to determine the bacterial burden (Fig. 2A). The rest of the homogenate was then filtered through a 70-µm-pore-size cell strainer; the flowthrough was pushed through a 5-µm-pore-size syringe filter and collected in a 1.5-ml tube (Fig. 2B). An aliquot of the filtrate was then plated to determine the number of viable B. pertussis bacteria that passed through the filter. The filtration allowed for isolation of ∼50% of the viable bacteria from the infected lungs (data not shown) (30). To confirm that the filtration method allowed for detection of bacterial transcripts, we performed quantitative reverse transcription-PCR (qRT-PCR) analysis on B. pertussis RNA samples from *in vitro* cultures grown in Stainer-Scholte liquid medium (SSM) and from B. pertussis-infected NSG lung (Fig. 2C). We observed increased *cya* and *ptx* gene expression in bacteria for all of our NSG mice compared to levels in bacteria grown *in vitro* (Fig. 2C). Expression of *fhaB,* the major adhesin, was decreased *in vivo* compared to the level *in vitro*. The genes *cya* and *ptx* are classified as class 2 Bvg system genes (23), whereas *fhaB* is a class I gene. Class I genes are usually expressed at different phases than class II genes, which could explain why *cya, ptx,* and *fhaB* are differentially expressed in these samples. BP2497 was more highly expressed *in vitro* than *in vivo* (Fig. 2C).

### *In vivo* RNA-seq analysis of B. pertussis infecting the murine lung.

Libraries were generated, and Illumina sequencing was performed on B. pertussis *in vitro* (SSM broth) and *in vivo* (filtered NSG mouse lung) RNA samples. As expected, the *in vitro* samples resulted in mapping of more than 90% of the reads generated. We were able to map, on average, 948,281 2- by 150-bp reads per biological sample, resulting in ∼1% of the reads corresponding to the pathogen. We obtained 71-fold more RNA mapping with our filtration method than in our initial attempts to perform dual sequencing on B. pertussis infecting the NSG lung (without filtration) (30). Overall, approximately ∼1 million reads from each *in vivo* sample mapped to the reference genome. Reads mapped to 94% of the genes on the Tohama I reference genome, giving sufficient data to describe the transcriptome. With this read depth, we observed that 83% of the genes of B. pertussis were expressed *in vitro* and that 89% of the genes were expressed *in vivo*. These numbers are similar to those obtained in our previous studies with the pathogen P. aeruginosa, for which we detected the expression of 98.2% of the genes in the lung during infection (29). Due to the large difference in the total numbers of mapped reads between *in vitro* and *in vivo* samples, we down-sampled the *in vitro* reads to 1 million to reduce the chances of overestimation errors generated by comparison of large to small numbers of reads. Down-sampling the reads resulted in the same ratios of reads from one gene to the next and allowed for a more appropriate analysis with less saturation effects due to the excess data depth of the *in vitro* samples.

### *In vivo* expression of known virulence-associated genes and virulence-repressed genes.

**FIG 3:**
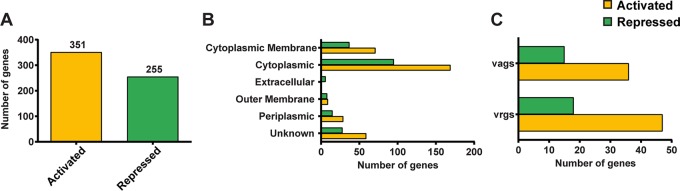
Systemic analysis of genes differentially expressed between *in vivo* and *in vitro* growth conditions of B. pertussis. (A) Total number of genes statistically activated or repressed in a comparison of B. pertussis growing in SSM (*in vitro*) to B. pertussis growing in NSG mice. (B) Predicted cellular localization of differentially expressed genes, according to the color scheme of panel A. (C) Numbers of virulence-activated genes (VAGs) and virulence-repressed genes (VRGs) *in vivo* compared to numbers *in vitro* revealed low correlation with Bvg designations. B. pertussis VAGs and VRGs were determined based on the study performed by Moon et al. (26).

We observed that the changes in the expression levels of 606 genes were statistically significant when *in vitro* and *in vivo* conditions were compared (Fig. 3A; see also Table S1 in the supplemental material). Overall, 351 genes were significantly activated, 255 genes were significantly repressed, and expression levels of 3,250 genes were not statistically changed between the *in vivo* and *in vitro* conditions (Fig. 3A). To understand the function of the genes with significant changes in expression, we separated them according to the subcellular localization of the proteins they encode (Fig. 3B). We observed that the products of the majority of genes up- and downregulated *in vivo* were predicted to be localized either in the cytoplasm or associated with the cytoplasmic membrane.

10.1128/mSphereDirect.00154-19.1 TABLE S1 *In vivo* RNA-seq analysis data set. RNA-seq was performed using CLC Genomics Workbench, version 9.5.4. All reads were trimmed based on quality scores with standard settings. All reads were mapped to the Bordetella pertussis Tohama I reference genome. Only paired reads were counted towards expression analysis. The following settings were used for RNA-seq analysis. Total count filter cutoff = 5.0. Estimate tagwise dispersions = Yes. Comparisons = All pairs. Bonferroni corrected = Yes. FDR corrected = Yes. The context table of the Excel file indicates the sample names, comparisons, as well as the raw and significant differentially expressed genes. The data set includes all gene expression data, subsets of activated and repressed gene sets, correlations with the van Beek et al. data set (28), and smaller systems of genes that were specifically described in the text in Fig. 5 and 6. All of the mapped reads are available at the Sequence Read Archive (SRA) under BioProject PRJNA433887 and SRA SRX3691783. Download Table S1, XLSX file, 2.9 MB. Copyright © 2019 Wong et al. 2019 Wong et al. This content is distributed under the terms of the Creative Commons Attribution 4.0 International license.

**FIG 4:**
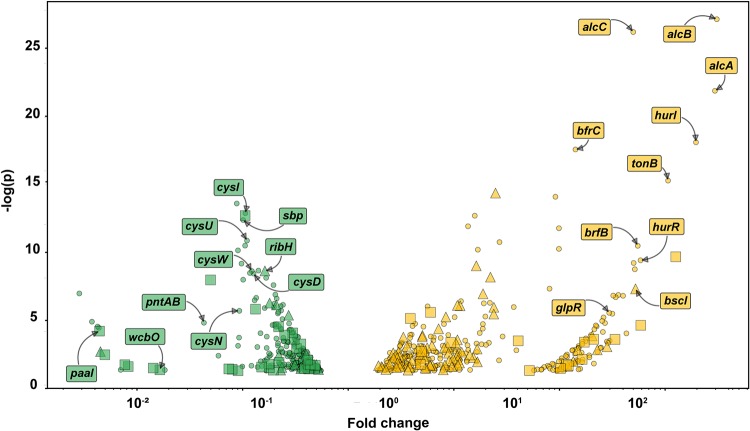
Volcano plot indicating B. pertussis gene expression in the infected lung compared to expression *in vitro.* Yellow denotes significant activated genes, and green indicates significant repressed genes. Virulence-activated genes (VAGs) are denoted by triangles and virulence-repressed genes (VRGs) are denoted by squares. Genes that are independent of the BvgAS system are shown with circles. B. pertussis VAGs and VRGs were determined based on the study performed by Moon et al. (26).

The *Bordetella* virulence gene system (BvgAS) controls expression of most of the virulence factors of B. pertussis. Based on the assumption that virulence genes should be activated *in vivo*, we classified them according to the predicted Bvg class they are associated with. Genes controlled by the BvgA transcription factor are referred to as virulence-activated genes (VAGs) or virulence-repressed genes (VAGs). In our study, we designated genes VAGs and VAGs according to an RNA-seq study on the Bvg regulon in B. pertussis performed by Moon et al. (26). Interestingly, we did not observe an absolute correlation between VAGs and VAGs *in vivo* (Fig. 3C); in fact, there was very little correlation observed. We predicted that most virulence-activated genes would be activated and that most virulence-repressed genes would be repressed *in vivo* compared to their expression under *in vitro* conditions. On the contrary, we observed that genes designated as activated during virulence were repressed *in vivo* and vice versa (Fig. 3C and 4). From these data, we hypothesize that the bacteria harvested from the lung for this study could be in several virulence phases but that our *en masse* analysis is not capable of distinguishing the individual phases. Future studies with single-cell analysis or protocols with lower numbers of bacteria may be able to tease out these differences.

### Systems-based analysis of differentially expressed genes.

**FIG 5:**
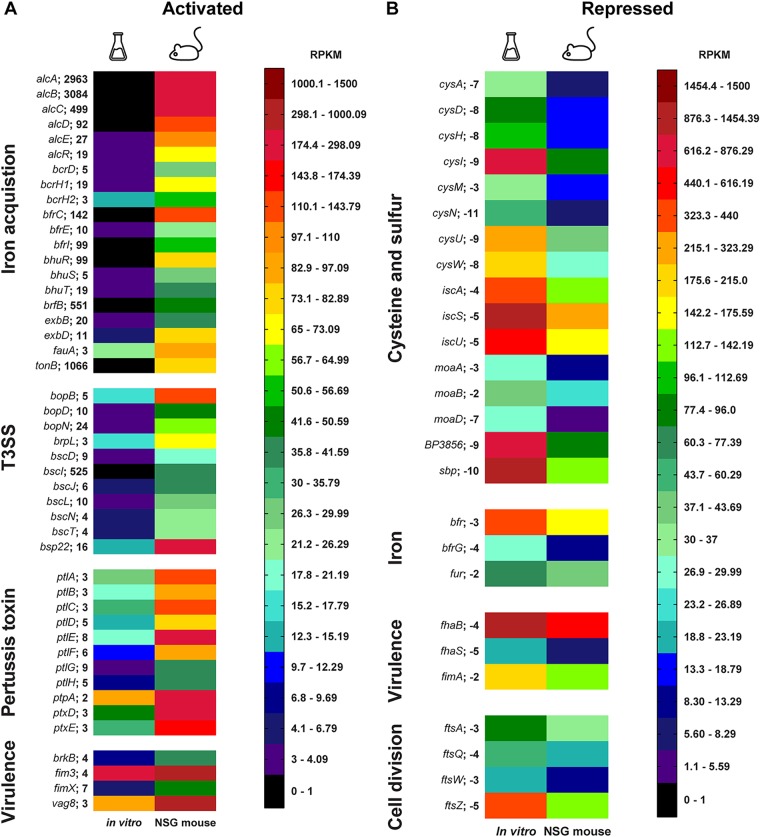
Heat map visualization of *in vivo*-activated and -repressed genes organized by function. The average number per million mapped reads (RPKM) is denoted by the color scale. The maximum value of the scale is set at 1,500 RPKM (red), and the minimum is at 0 RPKM (black). The distribution of the color scale of 24 colors is divided by the total number of data points per range. The Erlenmeyer flask represents *in vitro* conditions, and the mouse represents *in vivo* conditions. (A) Activated groups *in vivo* compared to expression *in vitro* include iron acquisition, type III secretion system (T3SS), pertussis toxin, and other virulence factors. (B) Repressed groups *in vivo* compared to expression *in vitro* include cysteine and sulfur, cell division, iron, and virulence factors. The average fold change *in vivo* compared to that *in vitro* is noted on the right of the gene name, and all genes shown were statistically significant.

We used Gene Ontology (GO) term and STRING analysis to group genes of related functions and plotted expression differences within a heat map (Fig. 5). In the heat map visualization, gene expression data are represented by the average number of reads per kilobase million (RPKM) for each biological sample. The heat map is composed of a 24-color scale, with each color representing a range of RPKM values evenly distributed across the total represented data set of expression values. To generate the color map, we divided each color across a range based on the number of total data points. This allowed for more distinct visualization for data ranges with more total data points. Four groups of genes of interest were activated *in vivo*, i.e., iron acquisition, type III secretion system, pertussis toxin, and secretion, as well as several key virulence genes (Fig. 5A). We observed that genes involved in cellular respiration, cell division, cell wall synthesis, and sulfur metabolism were repressed *in vivo*. Since genes involved in growth of B. pertussis were repressed *in vivo*, we hypothesize that the bacteria were growing more slowly *in vivo* than under *in vitro* growth conditions. Alternatively, *in vivo* metabolism may be altered by nutrient availably. Also, it could be hypothesized that *in vivo* growth is a combination of slower growth and altered metabolism. Interestingly, we observed that the *fhaB* gene encoding filamentous hemagglutinin was also repressed *in vivo* compared to its expression *in vitro* (Fig. 5B). We also observed lower *in vivo* expression of *fhaB* by qRT-PCR (Fig. 2C).

### *In vivo* B. pertussis activates expression of iron acquisition genes.

**FIG 6:**
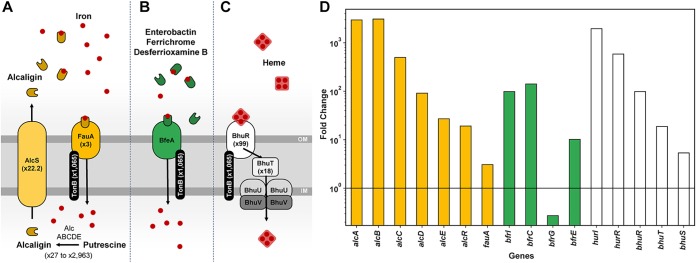
B. pertussis activates expression of iron acquisition systems *in vivo*. (A to C) A model representing B. pertussis alcaligin, enterobactin, and heme acquisition systems. OM/IM, outer/inner membrane. (D) B. pertussis iron and heme acquisition systems highly expressed *in vivo* compared to expression *in vitro*. Yellow denotes highly expressed B. pertussis alcaligin acquisition system genes, green denotes highly expressed enterobactin acquisition system genes, and white represents highly expressed heme acquisition system genes.

B. pertussis is known to sequester both iron and heme using nonredundant systems to support its need for iron during infection. B. pertussis uses both its own siderophore alcaligin and the xenosiderophores enterobactin (31), ferrichrome, and desferrioxamine B (32) produced by other bacterial species. Alcaligin is synthesized by various enzymes encoded by the *alcABCDE* operon and is exported by the permease AlcS (Fig. 6A). Once outside the cell, alcaligin binds iron. Ferric alcaligin interacts with, and is taken up by, the TonB-dependent outer membrane receptor FauA. B. pertussis also encodes the BfeA receptor that interacts with BfeB to take up xenosiderophores (Fig. 6B). Heme binds to the receptor BhuR on the surface of B. pertussis. The heme is then transported into the periplasm by a TonB-dependent mechanism, and the proteins BhuU and BhuV translocate heme to the cytoplasm (Fig. 6C). Several studies have shown that B. pertussis relies on the iron uptake systems for growth *in vivo* (33) and that iron starvation is a key host environmental cue for adaptation and virulence (34, 35). Interestingly, we observed that iron acquisition genes were highly activated *in vivo* (Fig. 6). Specifically, genes involved in alcaligin siderophore biosynthesis (*alcABCDER*), alcaligin siderophore receptor (*fauA*), xenosiderophore receptor (*bfeA*), and heme acquisition proteins (*bhuR*, *hurI*, *hurR*, *bhuT*, and *bhuS*) were significantly upregulated *in vivo* (Fig. 5 and 6D). These data support the idea that acquisition of iron is important for infection of B. pertussis in the host.

## DISCUSSION

The goal of this study was to characterize the *in vivo* transcriptome of B. pertussis during infection of the murine lung. qRT-PCR and microarray technologies have provided high-quality data concerning the gene expression of B. pertussis under various conditions, and microarrays have also been used to study the *in vivo* transcriptome (28). In this study, we aimed to build upon our past RNA-seq analysis (29, 30, 36) and to develop a protocol for isolating bacteria from the infected murine lung to perform RNA-seq and better understand the gene expression profile in the host. Using filtration, we were able to separate bacteria from the infected lung cell suspension. A total of 606 genes were differentially expressed *in vivo* versus expression *in vitro*. Remarkably, our analysis indicates that 84% of genes encoded in the B. pertussis genome are expressed at similar levels *in vitro* and *in vivo* in our comparison of growth in SSM broth and that in the mouse lung environment. These data are surprising as they indicate that more genes are expressed at similar levels under both conditions than are significantly differently expressed, suggesting that growth in SSM broth results in gene expression similar to that *in vivo*. In other studies, we have also compared growth in SSM to that in BG agar, and principal-component analysis indicates that SSM and BG agar induce distinct expression profiles (unpublished data). The genes most highly activated *in vivo* encode components of the iron and heme acquisition systems. Prior to our study, it was evident that iron is required for growth and virulence of B. pertussis. One of the factors that could account for this difference is that the SSM broth is rich in iron to allow optimal growth of the pathogen (37). B. pertussis is hemolytic due to ACT (38), which would lead to lysis of red blood cells, facilitating heme uptake (39). We also observed pertussis toxin and secretion genes to be highly expressed (Fig. 5A); but these data are not surprising because PT is a critical virulence factor for B. pertussis, and it is controlled by the BvgAS system as a virulent-phase gene (40). Also, it is not surprising to see iron acquisition upregulated in correlation with virulence gene expression since iron is an essential element that pathogens need for infection. On the other hand, we had expected that ACT would also be more highly expressed *in vivo*, but its expression during infection was not different from that in *in vitro* SSM growth. The type III secretion system of B. pertussis has been shown to be active in clinical isolates (41), and expression of the effector protein Bsp22 has been detected during the course of mouse infection (42). We observed many genes associated with the type III section system to be highly expressed *in vivo* during murine infection (Fig. 4). The *btrA* and *brpL* genes encode an anti-sigma factor and sigma factor system that control expression of the type III secretion system (24), and we also observed high expression of these genes *in vivo* (see Table S1 in the supplemental material). All together, these data suggest that that the type III secretion system is more highly expressed *in vivo* than *in vitro*.

van Beek and colleagues used RNA amplification and microarray analysis to determine *in vivo* gene expression of B. pertussis strains B1917 and B1920 in 6- to 8-week-old BALB/c mice (28). In their studies, bacteria were isolated at 7 days postchallenge from the nasal and bronchoalveolar lavages. The study revealed enhanced gene expression of fimbrial adhesion genes, type III secretion system genes, *btrA* and *brpL* (anti-sigma and sigma factor), pertussis toxin, dermonecrotic toxin, and other genes. Due to the differences in detection technologies (microarray and RNA-seq), we did not directly compare the relative gene expression fold changes, but we correlated gene expression as activated or repressed between the two data sets. When we compare our study to that of van Beek et al., we see strong correlations with several key differences. We found 111 genes to be commonly activated in both sets of data, and these included the virulence factors already described above, as well as many hypothetical genes (Table S1). There were 16 commonly repressed genes *in vivo*, which included the cysteine operon. The *cysAWTP* genes encode proteins involved in sulfate/thiosulfate import. When our activated *in vivo* gene expression list was compared to the activated gene expression list of van Beek et al., there was a noticeable set of genes that were different, and most corresponded to iron acquisition genes. For *in vitro* cultures, van Beek et al. used THIJS medium (43) which has a similar amount of supplemented iron as SSM but different nitrogen and carbon ratios. In addition to use of a slightly different liquid broth growth medium, 0.2 mg/ml heptakis-cyclodextrin was supplemented to enhance growth. Heptakis is added to liquid growth medium to ameliorate the effects of fatty acids on the growth of B. pertussis (44–48). For our growth conditions, we utilized fresh, never-used glassware, which negates the need to add heptakis to stimulate growth (unpublished observation). Other differences between these two studies are the mouse strains (NSG versus BALB/c) and challenge strains of B. pertussis that were used. We used NSG mice in order to get sufficient amounts of bacteria for RNA-seq. It is possible that these aforementioned experimental design differences account for the differences between results of the *in vivo* microarray and our *in vivo* RNA-seq study. Overall, the two studies agree in the majority of observations and advance our knowledge of the infection transcriptome of B. pertussis.

In this study, we infected NSG mice to achieve a higher bacterial burden in the lungs to characterize the infection transcriptome of B. pertussis using RNA-seq. By using NSG mice, we subjected B. pertussis to an immune environment lacking mature lymphocytes, functional macrophages, and dendritic cells. The absence of these cells could change the transcriptome of B. pertussis during infection because the bacteria are not encountering immune effector mechanisms to clear the infection, thus potentially changing the activation and repression of certain virulence factors. This is a caveat of our study, and in the future, we will aim to perform *in vivo* RNA-seq in immunocompetent mice once we overcome several technical hurdles. Another caveat that should be considered is the high bacterial burden. It is not likely that B. pertussis grows to high density within the human host; therefore, bacterial cell density could affect total gene expression, and future studies will aim to analyze smaller populations of B. pertussis from the host.

*In vivo* RNA-seq is in its early stages of development, and as of the writing of the manuscript, there are approximately 20 total publications that have profiled the transcriptomes of bacteria during infection of host animals or host cells (49). All *in vivo* RNA-seq projects must overcome the low abundance of pathogen RNA due to the large amounts of host RNA (49, 50). In a recent RNA-seq analysis of Yersinia pseudotuberculosis-infected mouse Peyer’s patches, only 0.2 to 1.4% of reads corresponded to pathogen RNA. Despite that small amount of RNA read data, important observations were made concerning *Yersinia* pathogenesis (51). At the onset of our study, we aimed to map the *in vivo* transcriptome of B. pertussis in the murine host using RNA-seq. While we were able to overcome the hurdle of isolating bacteria from the infected lung, we still managed to retain a large amount of RNA from the host (98 to 99% of the read data). Remarkably, the majority of the host RNA corresponds to mitochondrial RNA (data not shown). During filtration, we hypothesize that the small size of mitochondria allowed them to flow through the filter. We filtered mouse lungs with the 5-µm-pore-size filter and used microscopy to observe that mitochondria were in high abundance in the lung filtrate (data not shown). While RNA-seq provided us with sufficient data to describe a strong draft transcriptome, we now aim to advance toward determining gene expression from smaller populations of B. pertussis in the respiratory tract. We hypothesize there are multiple populations in any infected host, such as Bvg^+^, Bvg^−^, and Bvg^i^ B. pertussis. One caveat of our approach is that some bacteria do not go through the filter. We are able to increase our total recovery by flushing more phosphate-buffered saline (PBS) through the filter. But it is likely that the bacteria that do not pass through the filter are in microcolonies or are attached to cells. In order to characterize these populations, we will need to develop protocols to isolate small populations of bacteria with extremely minimal amounts of host RNA contamination. We ultimately want to develop a protocol and work flow that would allow for analysis of strains directly from infected patients or other model organisms. Our future studies will also aim to utilize immunocompetent hosts to observe the effects of the innate and adaptive immune systems on B. pertussis during infection. There are several technologies for eukaryotic cells that allow for single-cell RNA-seq analysis (52). A significant amount of protocol development will need to occur for that to be possible for bacteria. We envision that in the coming decade single-cell bacterial RNA-seq will become the standard, and it will be interesting to compare single-cell data to data reflecting the average of all infecting bacteria, such as the data set we present in this study. It is possible that there are bacteria in different phases of virulence, but technological improvements in the work flow are still required. As we learn more about what these pathogens are expressing during infection, it is likely that new antigens that were previously not identified could be exploited for vaccine development. In the near future, it may be possible to isolate pathogens directly from human patients and determine the human-specific and phase-specific (initial infecting or transmitting) transcriptomes of the pathogen. We aim to fully characterize the transcriptome of the human pathogen B. pertussis and to use *in vivo* RNA-seq to enhance our knowledge of how pathogens infect their hosts.

## MATERIALS AND METHODS

### Bacterial strains and growth conditions.

B. pertussis strain UT25 (UT25Sm1) (53) was cultured on Bordet-Gengou (BG) agar (Remel) (54) supplemented with 15% defibrinated sheep blood (Hemostat Laboratories) for 48 h at 36°C. B. pertussis was then transferred from BG plates to three flasks containing 12 ml of modified Stainer-Scholte liquid medium (SSM) (37). SSM cultures were not supplemented with cyclodextrin [heptakis(2,6-di-O-methyl)-β-cyclodextrin)] and were grown for ∼22 h at 36°C with shaking at 180 rpm until the optical density at 600 nm (OD_600_) reached 0.5 on a 1-cm-path-width spectrophotometer (DU 530; Beckman Coulter). For infection of NSG mice, cultures were then diluted to provide a challenge dose of 2 × 10^7^ CFU in 20 μl. For growth of Pseudomonas aeruginosa strain PAO1, *Pseudomonas* Isolation Agar (Difco) was used. Bordetella bronchiseptica RB50 and Escherichia coli TOP10 were cultured on lysogeny agar (10 g of NaCl, 5 g of yeast extract, 10 g of tryptone) at 36°C for 18 h. *In vitro* cultures of bacteria were grown and then filtered through 5-µm-pore-size syringe filters (Minisart; Sartorius). The total number of input and output bacteria were determined through serial dilutions and plate counts on the appropriate medium, and the percent recovery of each species was determined.

### Murine B. pertussis challenge.

NSG mice (NOD.Cg-*Prkdc^scid^ Il2rg^tm1Wjl^*/SzJ, stock number 005557; Jackson Laboratory) were raised in-house by the West Virginia University (WVU) Transgenic Animal Core Facility. Mice were anesthetized by intraperitoneal injection of ketamine and xylazine in saline according to approved protocols. Two 10-μl doses of B. pertussis were pipetted directly into each nostril of the mouse to provide a challenge dose of 2 × 10^7^ CFU in 20 μl. Five to seven mice were infected with strain UT25, and mice were euthanized by pentobarbital injection 3 days postchallenge. All murine infection experiments were performed according to protocols approved by the West Virginia University Institutional Animal Care and Use Committee (IACUC; protocol numbers 14-1211 and 1602000797), conforming to AAALAC accreditation guidelines.

### Isolation of B. pertussis from infected NSG lungs.

Lungs were extracted and quickly homogenized in a sterile Dounce glass homogenizer. A sample of the homogenate was used to determine total bacterial burden by performing serial dilutions in PBS that were then plated on BG medium containing streptomycin (100 μg/ml). The rest of the homogenate was passed through a 70-µm-pore-size cell strainer (VWR), and then the filtrate was passed through a 5-µm-pore-size filter (Minisart type 17594 cellulose acetate; Sartorius) via a syringe. A sample of the final filtrate was used to determine the number of bacteria recovered. The filtrate was then centrifuged (4°C at 16,100 × *g*) to pellet the bacteria. The supernatant was removed, and 200 µl of bacterial RNAprotect was added to the pellet. The pellet was resuspended and then pelleted by centrifugation for 2 min at 16,100 × *g*. The pellet was then frozen at −80°C until RNA isolation.

### RNA isolation.

One B. pertussis-infected NSG mouse was used per biological sample for RNA-seq. RNAsnap lysis was used to isolate RNA from the filtered B. pertussis cells from the infected murine lungs (55). Cell pellets were resuspended in RNAsnap extraction solution (18 mM EDTA, 0.025% SDS, 1% β-mercaptoethanol, 95% formamide) by vortexing vigorously. Samples were incubated at 95°C for 7 min and pelleted by centrifugation at 16,100 × *g* for 5 min at room temperature. The supernatant containing RNA was then pipetted into a fresh tube without disturbing the clear gelatinous pellet, and DNA and RNA concentrations were measured using a Qubit 3.0 fluorometer (ThermoFisher). The resulting RNA was treated with RNase-free DNase (Qiagen). To remove the DNase, the samples were then cleaned up on an RNeasy Mini column (Qiagen). The resulting RNA was quantified on a Qubit 3.0 fluorometer. To ensure that RNA was DNA free, 25 ng of RNA was checked by quantitative PCR (qPCR) amplification as described below and was used only for cDNA if the threshold cycle (*C_T_*) was >30. The following primers were used for the DNA-free confirmation: mouse gapdh-F (CATGGCCTTCCGTGTTCCT), mouse gapdh-r (GCGGCACGTCAGATCCA) and fhaBF/fhaBR (25). The RNA integrity was assessed by running the samples on an Agilent BioAnalyzer RNA Pico chip. To compare *in vitro* to *in vivo* samples, B. pertussis was cultured in SSM broth, and bacterial RNA was isolated with RNAsnap. The RNA was subjected to DNase treatment (Qiagen), and cleanup procedures were performed as described above.

### qRT-PCR.

For quantitative reverse transcription-PCR (qRT-PCR) analysis, cells stored at −80°C in RNAprotect (Qiagen) were lysed using the RNAsnap method as described above (55). To ensure that RNA was DNA free, 25 ng of RNA was checked by qPCR amplification as described below and was used only for cDNA if no amplicon was observed and if the *C_T_* value was >30 using the fhaBF and fhaBR primers. cDNA was synthesized using Moloney murine leukemia virus (M-MLV) reverse transcriptase (Promega) per the manufacturer’s instructions using 200 ng of RNA and gene-specific reverse primers for targets. Twenty-five microliter qPCRs were set up with Excella SYBR Green PCR master mix (Worldwide Medical Products), per the manufacturer’s instructions, using 1 μl of cDNA that was generated from 500 ng of RNA. A minimum of three technical replicate reactions were run per gene target per sample on a Step One Plus qPCR thermocycler (Applied Biosystems). Primers were designed on Primer3 (Primer-BLAST; NCBI) and checked for specificity by PCR. Melt curve analysis as well as subsequent agarose gel electrophoresis was performed on all reaction products. Gene expression was normalized to that of the *rpoB* reference using the 2^−ΔΔ^*^CT^* method (56). For statistical analysis, the Δ*C_T_* for the three biological replicate experiments was calculated, and a Student's *t* test was performed. Standard error of mean was calculated based on the variability of the Δ*C_T_* value of three biological replicates. The following primer sequences used in this study were described by Bibova et al. (57): cyaF (CGAGGCGGTCAAGGTGAT), cyaR (GCGGAAGTTGGACAGATGC), ptxAF (CCAGAACGGATTCACGGC), ptxAR (CTGCTGCTGGTGGAGACGA), bvgAF (AGGTCATCAATGCCGCCA), bvgAR (GCAGGACGGTCAGTTCGC), fhaBF (CAAGGGCGGCAAGGTGA), fhaBR (ACAGGATGGCGAACAGGCT), rpoBF (GCTGGGACCCGAGGAAAT), and rpoBR (CGCCAATGTAGACGATGCC). Primers for the amplification of BP2497 were also designed in a previous study: BP2497F (TCGGATCGCACCAATTACTTC) and BP2497R (CCTTGGCGATCAGCGAGTT) (25). The following primers were used for glyceraldehyde-3-phosphate dehydrogenase (GAPDH): gapdhf (CATGGCCTTCCGTGTTCCT) and gapdhr (GCGGCACGTCAGATCCA).

### Library construction and Illumina sequencing.

*In vitro* and *in vivo* RNA samples were subjected to both bacterial and murine RiboMinus Transcriptome Isolation kits (ThermoFisher), in order to deplete rRNA. The RNA depleted of rRNA was then converted into Illumina sequencing libraries with a SMART-Seq, version 4, low-input kit (Clontech). Resulting libraries passed standard Illumina quality control PCR and were sequenced on an Illumina Hiseq 4000 at Admera Health (South Plainfield, NJ). A total of 90 to 100 million 2- by 150-bp reads were devoted to each sample, both *in vitro* and *in vivo*.

### RNA-seq and bioinformatics analyses.

The reads were aligned to the B. pertussis Tohama I genome (58) using CLC Genomics Workbench, version 9.5 (Qiagen). Table S1 in the supplemental material contains the gene expression data obtained for this project. The number of reads per kilobase per million (RPKM) and fold change for each gene were calculated. Fold change was calculated by comparison of the number of *in vitro* UT25 B. pertussis reads to the number of *in vivo* NSG mouse UT25 reads. Based on the number of reads mapped to the genome of UT25 *in vivo*, we down-sampled the total *in vitro* reads to 1 million 2- by 150-bp reads to compare similar numbers of reads between the *in vitro* and *in vivo* data sets. Down-sampling was performed in CLC Genomics, version 9.5 (Qiagen), utilizing the random output, which returns a random sample of the reads, resulting in an equal distribution to the original total read data set. An empirical analysis of digital gene expression (EDGE) was performed to determine differentially expressed genes (59). Genes with an EDGE test *P* value of ≤0.05 were considered differentially regulated. Genes were annotated with custom Gene Ontology Annotation (GOA) files that were created from annotations downloaded from UniProt (proteome identification number UP000002676). Subsets of statistically significant genes were compared to the total genes of the genome to identify GOA terms that were enriched using a HyperG test and to determine the most common and active biological processes, cellular components, and molecular functions in the experiment (60). A volcano plot was created by plotting significant genes using their EDGE test fold change values and the negative log of their EDGE test *P* values (59). The STRING functional protein association networks database (61) was used to associate systems of genes in order to create a heat map visualization for activated and repressed *in vivo* genes.

### Accession number(s).

The mapped reads are available at the Sequence Read Archive (SRA) under BioProject accession number PRJNA433887 and SRA accession number SRX3691783.
